# Diagnostic and Research Aspects of Small Intestinal Disaccharidases in Coeliac Disease

**DOI:** 10.1155/2017/1042606

**Published:** 2017-04-20

**Authors:** Tanja Šuligoj, Paul J. Ciclitira, Borut Božič

**Affiliations:** ^1^King's College London, Gastroenterology, Rayne Institute, St Thomas' Hospital, London SE1 7EH, UK; ^2^Faculty of Pharmacy, University of Ljubljana, Aškerčeva 7, SI-1000 Ljubljana, Slovenia

## Abstract

Disaccharidases (DS) are brush border enzymes embedded in the microvillous membrane of small intestinal enterocytes. In untreated coeliac disease (CD), a general decrease of DS activities is seen. This manuscript reviews different aspects of DS activities in CD: their utility in the diagnosis and their application to in vitro toxicity testing. The latter has never been established in CD research. However, with the recent advances in small intestinal organoid techniques, DS might be employed as a biomarker for in vitro studies. This includes establishment of self-renewing epithelial cells raised from tissue, which express differentiation markers, including the brush border enzymes. Determining duodenal DS activities may provide additional information during the diagnostic workup of CD: (i) quantify the severity of the observed histological lesions, (ii) provide predictive values for the grade of mucosal villous atrophy, and (iii) aid diagnosing CD where minor histological changes are seen. DS can also provide additional information to assess the response to a gluten-free diet as marked increase of their activities occurs four weeks after commencing it. Various endogenous and exogenous factors affecting DS might also be relevant when considering investigating the role of DS in other conditions including noncoeliac gluten sensitivity and DS deficiencies.

## 1. Introduction

In some individuals, gastrointestinal symptoms including diarrhoea are related to the ingestion of certain forms of dietary carbohydrate. Symptoms are attributable to one or multiple enzyme deficiencies of the small intestinal mucosa that hydrolyse disaccharides [1]. These include lactase deficiency (congenital and adult type), sucrase-isomaltase deficiency, maltase-glucoamylase deficiency, and trehalase deficiency. With the exception of adult lactase deficiency, other deficiencies listed above are relatively rare [2, 3]. Other conditions, including coeliac disease (CD), which result in small intestinal injury may cause reduction of disaccharidase (DS) activities. It has also been suggested that secondary DS deficiencies may possibly account for symptoms in patients with CD who have intact villi [4].

Lactose which is digested by the brush border enzyme lactase is one of the poorly absorbed short-chain carbohydrates, often referred as FODMAPs (fermentable, oligo-, di-, monosaccharides and polyols). It has been shown that reduced FODMAP intake improves gastrointestinal symptoms in patients with noncoeliac gluten sensitivity (NCGS) [5]. It has been demonstrated that components other than gluten in wheat might be important in NCGS, namely amylase-trypsin inhibitor [6, 7]. Lack of biomarkers for this condition [6] might prompt researchers to investigate the potential role of brush border enzymes such as lactase.

In untreated CD, a general decrease of DS activities is seen [8–11]. Before the advent of serological testing, small intestinal DS activities were important laboratory parameters for aiding the diagnosis of CD. Determination of individual DS activity has also been crucial for the differential diagnosis of inborn metabolic disorders. Measurement of their activity aids distinction between primary and secondary DS deficiencies [10, 12, 13]. Primary types of DS deficiencies constitute “inborn errors of metabolism” of a specific enzyme [3, 14]. An example of this is congenital lactase deficiency, in which there are normal levels of maltase and sucrase with reduced lactase. CD is a secondary DS deficiency as all DS are reduced due to gluten-induced injury to the small intestinal mucosa [8, 14].

Marked increase of DS activities occurs four weeks after commencing a gluten-free diet (GFD), but their activities are again reduced if coeliac patients revert to a normal diet [11, 15]. In patients with treated CD, intraduodenal instillation of gluten produces characteristic histological changes and an associated marked reduction in disaccharidase activities within 3.5 hours [16]. The reduction of DS activities correlates with the histological grade of the biopsy [11, 17, 18] such that DS could be employed as a biomarker of CD.

This manuscript reviews different aspects of DS activities in CD: their utility in the diagnosis and their application to in vitro toxicity testing. Furthermore, we describe recent advances in small intestinal organoid techniques, including coculture with immune cells, which offer an exciting opportunity to develop state-of-the-art in vitro models for CD research. In this model, brush border enzymes could be employed as markers of CD pathogenesis.

## 2. Diagnostic Aspects of Small Intestinal Disaccharidase Activities in CD

The gold standard for the diagnosis of CD and following the effect of GFD is the examination of duodenal biopsies in conjunction with CD serology including tissue transglutaminase. Measurement of DS activities provides additional information at the time of diagnosis and during follow-up to assess the response to a GFD [19]. Furthermore, it has been shown that determining DS activities might aid the diagnosis of CD where milder histological changes are seen, including Marsh I and II [20] abnormalities in small intestinal biopsies [4, 10, 18].

### 2.1. Positive and Negative Predictive Values of Mucosal Villous Atrophy

The measurement of brush border enzyme activities offers an additional objective tool to evaluate the severity of the histological abnormalities in untreated coeliac patients [11]. Duodenal DS activities are good predictors of the grade of mucosal villous atrophy in CD. Positive predictive values for moderate or severe villous atrophy are as follows:

- 90% for maltase (maltase activity < 150 U/g protein)
- 86% for sucrase (<40 U/g protein)
- 71% for lactase (<20 U/g protein).

Decreased maltase and sucrase activities thus have a high positive predictive value for the degree of mucosal villous atrophy. Predictive values of lactase activity are lower, probably due to the presence of primary lactase deficiency in coeliac patients. No patient with DS activities in the normal range exhibited severe villous atrophy [19].

### 2.2. Mucosal Healing in CD with a Gluten-Free Diet (GFD)

It is well established that brush border enzyme activities are reduced in untreated coeliac patients. However, the activities recover during remission, notably four weeks after commencing a GFD [11]. It is important to note the different responses between the DS and a GFD; marked increase of alpha glucosidases activities occurs, whereas lactase activity remains low in many patients [8]. Peña et al. demonstrated that response of lactase to a GFD is variable, sometimes showing full recovery in a few months, but sometimes remaining depressed for years [9]. The age of the patient is of great importance in the rate of recovery of lactase activity. Patients aged less than 30 years usually show full recovery in the course of a few months, whereas most of the older patients show little or no recovery in this space of time [9]. Persistence of low lactase activities despite good histological improvement has been demonstrated in other studies for some patients, particularly adults [14, 19, 21].

With the exception of lactase [8], the measurement of DS may provide a quantitative index of improvement of the small intestinal mucosa [21]; their increase correlates well with the recovery of the mucosa based on small intestinal biopsy histology [19]. In addition to histological and serological follow-up, measurement of DS activities offers an additional potential tool to assess treatment of CD with a GFD [19]. Sucrase activity is the best indicator of the mucosal response to a GFD. However, it is interesting to note that even after two years' treatment of CD with a GFD, sucrase activity in the distal duodenum does not increase to the level of controls, although clinical effects are observed earlier [22]. Generally, enzyme activities in the small intestinal mucosae of patients with CD in remission are lower than those of control groups matched for age, sex, and site of biopsy [21]. However, coeliac patients on a GFD have significantly increased levels of maltase and sucrase but limited increase in lactase activity compared with an untreated group [14]. It is worthwhile mentioning that a strict GFD is the treatment of choice not only for CD but also for CD-associated secondary DS deficiencies [23].

### 2.3. Diagnosis of CD with Marsh I/II Score of Small Intestinal Mucosal Biopsies (Subclinical CD)

In small intestinal mucosal biopsies with a Marsh score of I and/or II, there is no villous atrophy [20]. Villous atrophy comprises only the end stage in the clinical course of the disease; CD develops gradually from small-bowel mucosal inflammation to crypt hyperplasia and finally to villous atrophy [24].

Milder biopsy changes that are limited to lymphocytic infiltration with or without crypt hyperplasia may require additional data in order to increase the certainty of a diagnosis of CD, as these histological changes are not exclusive to CD [4]. The diagnosis of CD may be enhanced by genotyping (HLA-DQ2 or HLA-DQ8); serological markers [4] and immunohistochemical staining of the *γδ*+ve intraepithelial lymphocytes found in patients with CD may also be used to confirm the diagnosis [24, 25].

Damage to the microvilli may be one of the earliest gluten-induced alterations in CD [4]. Therefore, biochemical changes can precede histological abnormalities of the duodenal biopsy observed by light microscopy [18]. Murray et al. reported diagnosing CD on repeat biopsy in 4 out of 37 (10.8%) patients who had no villous atrophy in their initial biopsy a few years earlier. However, these four patients had decreased DS activities and minor histological changes (Marsh I or II) in their initial biopsy. Reduced DS activities without villous atrophy may therefore represent early CD [18].

Mones et al. [4] observed a marked reduction of DS activities in paediatric patients with CD whose histological changes in their small intestinal biopsies scored as Marsh I or II (with intact villi). A positive test for predicting CD was defined by the following cut-off values for individual DS activity: lactase, ≤15 units/g protein; sucrase, ≤25 units/g protein; maltase, ≤100 units/g protein; and palatinase, ≤5 units/g protein. Diagnostic sensitivity ranged from 74% to 85%, with the highest sensitivity observed for lactase. Diagnostic specificity ranged from 57% to 91%, with the highest specificity for sucrase. The positive predictive value of a DS deficiency to predict CD ranged from 70% to 91%. Negative predictive values (a normal DS level) ranged from 72% to 76% to predict a biopsy not considered to be CD [4]. DS deficiency found in duodenal biopsies may therefore aid the diagnosis of CD in biopsies with intact villi [4]. A similar finding was reported in another study where evaluation of DS activities provided evidence to support diagnosis of CD in some patients [10].

## 3. Research Aspects of Disaccharidase Activities in Coeliac Disease

DS activities in small intestinal mucosal biopsies can be determined by the Dahlqvist method. The tissue is incubated with the relevant disaccharide and liberated glucose determined by colorimetric assay using TRIS-glucose oxidase reagent. Units of DS activity are expressed as micromoles of disaccharide hydrolysed per minute per gram of mucosa (wet weight) [26].

### 3.1. Factors Affecting Enzyme Activities

DS are reduced in active coeliac disease. They may be reduced in other conditions including inflammatory bowel disease, food allergy, dyspepsia, protein-energy malabsorption, immunodeficiencies, and infectious diseases (such as giardiasis, viral infections, and small intestinal bacterial overgrowth) [1, 18, 22, 27]. The decrease of DS activities can usually be reversed by successful treatment of the underlying disease. Interestingly, DS can also be increased abnormally in diabetic patients [28]. A reduction of individual DS deficiencies is seen in primary DS deficiencies which include congenital lactose intolerance, sucrase-isomaltase deficiency, maltase-glucoamylase deficiency, and trehalase deficiency [2, 3].

In healthy individuals, DS activities can show a broad range of absolute values [12, 29]. There are several endogenous and exogenous factors that affect their activities.

#### 3.1.1. Endogenous Factors

DS activities vary along the longitudinal axis (duodenum-jejunum-ileum) of the gut [19]. Lactase has maximum activities at 50–200 cm from the ligament of Treitz and is almost absent in distal ileum. Sucrase activities are constant along the small intestine. Maltase is twice as abundant in the distal ileum compared to that in the proximal jejunum [30].

Ethnicity affects DS values as demonstrated when comparing African and Finish children with normal villous architecture; the former had lower activities of duodenal lactase, sucrase, and maltase. About one-third of Finnish children exhibit lactase activity below the established reference range of 20 U/g protein, as opposed to two-thirds of African children [29].

Sex does not affect any of the DS activities [29]. Age has a significant effect on lactase activity only; it decreases with age. In some black children, lactase deficiency may develop after the age of 3 years and is not associated with mucosal disease [29].

Circadian rhythm influences DS activities [31, 32]. In addition, oscillations of DS activities correlate to the rhythm of food intake [33].

Patchy mucosal changes that can occur in CD affect the absolute values of DS activities found in biopsies. Jonsson et al. [34] demonstrated that there was approximately 30% coefficient of variation in DS activity of specimens taken from two sites in the duodenum.

#### 3.1.2. Exogenous Factors

Sucrose feeding is known to increase the activity of sucrase-isomaltase. The increase is a result of de novo synthesis of the enzyme which reaches its peak (2.6-fold increase) 12 hours after starting a sucrose-containing diet [31, 35]. Degradation of the enzyme is independent of the diet [31].

Fasting hampers intestinal epithelial cell renewal. It causes a reduction of small intestinal mass, villous size, and crypt enterocyte mitotic index. Carbohydrate deprivation induces a fall in intestinal sucrase which is restored by carbohydrate feeding [31].

The levels of DS also vary according to the site of biopsy [4]. This applies not only to the longitudinal axis (duodenum-jejunum-ileum) in the small intestine but also to the crypt-villous axis. Since DS are embedded in microvilli of villous enterocytes, their activity is dependent upon the number of villous enterocytes present in the biopsy [18], so that the more superficial biopsies having a higher epithelial content might have a higher DS content.

### 3.2. Ex Vivo and In Vitro Methods for Studying Gluten Toxicity in Coeliac Disease

There is no animal model reproducing all the features of coeliac disease [36]. In vivo testing is the gold standard for assessing coeliac toxicity [37, 38]. However, it is well established that in vivo administration of gluten can cause systemic injury to the patient. Therefore, in vitro methods are usually employed before in vivo studies are undertaken when assessing foodstuffs for lack of CD toxicity [39, 40].

The most reliable in vitro method is small-bowel mucosal biopsy culture [37] often referred to as duodenal biopsy organ culture (OC). The method was originally described by Browning and Trier [41]. There is significant requirement for preclinical in vitro studies, so the principle of Browning and Trier's method has been further developed and applied to testing probiotics that requires an apical stimulation of intestinal mucosal explants [42]. OC enables various other applications, for example, biochemical studies of synthesis and processing of DS [1, 43], dimeric assembly of DS [44], and studying effects of insulin on elevated DS activities in diabetic subjects [28].

Browning and Trier's original method has been modified so that the system detects harmful effects of gluten by adding it to culture medium and evaluating subsequent histological, morphological and immunological abnormalities. Modified Browning and Trier's technique is still being widely used in studies that examine CD pathogenesis [36] and in testing for coeliac toxicity [39, 40, 45–47]. The OC system has also been shown to be useful in aiding the diagnosis of CD, mainly in cases without villous atrophy or in seronegative patients [48].

#### 3.2.1. Early Work on Small Intestinal Organ Culture and the Study of Brush Border Enzymes in CD

Enterocyte damage is the hallmark of coeliac disease [49]. The toxic effect of gluten on small intestinal mucosa was demonstrated biochemically by measuring the activity of the brush border enzyme alkaline phosphatase (AP) [50] in OC. The activity of AP obtained from biopsies of untreated CD patients increased when the tissue was incubated in gluten-free medium. This increase was inhibited by the presence of gluten peptides in the culture medium demonstrating the toxic effects of gluten [50].

Katz and Falchuk [51] subsequently proposed the use of the small intestinal OC technique as a predictive test for the definitive diagnosis of gluten sensitivity. Twenty-two of 26 patients diagnosed with CD had shown gluten sensitivity in vitro on their initial biopsies. A rise in AP activity of intestinal tissue from these 22 patients was inhibited by the presence of gluten peptides in the medium. The false-negative rate for establishing the diagnosis of CD was therefore 15% (4 of 26). In their study, there were also 14 patients with abnormal mucosa who were shown not to have CD. Thirteen of them did not show gluten sensitivity in vitro (a false-positive rate of 7%). All patients with normal biopsies were classified correctly. These exciting results suggested that small intestinal organ culture could be used for the prospective diagnosis of CD. In another study, Falchuk et al. [52] again demonstrated gluten sensitivity in vitro in patients with active CD. They further suggested there were differences according to the subjects' histocompatibility type.

Other researchers undertook similar OC studies, measuring AP activity and another brush border enzyme *α*-glucosidase. Howdle et al. [53] and Hauri et al. [54] could not reproduce in vitro effects of gluten on duodenal biopsies from untreated CD patients nor by AP or *α*-glucosidase activity assessment.

Mitchell et al. [55] showed that there is a progressive loss of proteins from the tissue during OC. At the same time, the levels of brush border enzymes decrease and accumulate in the medium. They therefore suggested expressing enzyme activities as mU/ml of culture medium, as opposed to U/g protein, due to protein losses during organ culture and recovered enzyme activities in the medium [55].

Several authors investigated the proteins, the DNA content, and the brush border enzymes in small intestinal biopsy OC. Most have observed a decrease of all these parameters in cultured biopsies with the exception of the AP activity which increased in practically all of the cases [56]. DS are probably more readily lost into the medium during culture than AP [55, 57].

#### 3.2.2. Cell Models and Enzyme Activities

In addition to CD patient small-bowel mucosa OC, researchers have used different cell lines for in vitro studies of CD. Established cell models are based on gluten-sensitive T cell lines and clones isolated from individuals with CD, which are still being widely used in screening for CD toxicity [39, 58, 59]. Significant amounts of research have also been undertaken using epithelial cell lines of cancerous origin [36] which are relatively easy to grow. However, it is not established to what extent malignant transformation affects their possible dependence on surrounding microenvironmental influences. It has been shown that, unlike normal epithelial cells, colon cancer cell lines do not require addition of growth factors for their expansion in vitro [60]. In addition, Caco2 cells express considerably lower amounts of brush border enzymes in comparison to normal enterocytes [44].

Before Sato et al. [60] published their breakthrough discovery on intestinal organoids, enterocytes were notoriously difficult to grow in vitro. Over decades, many studies of normal gut epithelium have reported difficulties in maintaining culture of these cells for more than a few days ([61] and references therein) despite employing different isolation protocols and subsequent culture conditions. It was thought for many years that long-term cultures from primary human tissue could not be established unless the cells were genetically transformed. It was also discovered that disruption of the interactions between normal epithelial cells and surrounding extracellular matrix induces apoptosis of the cells [62].

Quaroni et al. [63], however, managed to establish a long-term culture of rat small intestinal epithelial cells. Their initial approach for isolation was that used in OC, but cultured epithelial cells had the features of undifferentiated small intestinal crypt cells. Poor differentiation levels of cultured enterocytes in vitro were reported in other studies using human, mouse, rat, and bovine intestinal biopsies [64, 65] which are consistent with our observations (unpublished data). Rusu et al. [64] studied differentiation in vitro by assessing specific activities of maltase and AP in bovine samples of (a) freshly scraped epithelia, (b) organoid suspensions used to seed cultures (not to be confused with Sato's organoids), and (c) intestinal cell cultures which include primary cultured cells and cells cultured after the first and second passage. Organoid suspensions presented a 50% reduction in regard to the fresh epithelium preparation. Maltase activity used as a differentiation marker clearly decreased in primary cultures and with subsequent culture passages toward a stable low level. Similar results were obtained from the measurements of the intestinal AP activity. These results reflected a loss of cell differentiation in vitro [64].

The epithelium is only one (i) of four components of an integrated functional unit which also consists of (ii) extracellular matrix, (iii) mesenchyme-derived cells, and (iv) luminal factors. Isolation of enterocytes from their mesenchymal environment results in loss of differentiation [66]. Nontransformed human foetal small intestinal cells grown on plastic have characteristics of undifferentiated crypt enterocytes [67]. The sequential addition of connective tissue and luminal molecules to nonmalignant human foetal enterocytes in vitro induced a spectrum of changes in the epithelial cell type toward full differentiation [66]. A rat small intestinal cell line also differentiated, as assessed by a significant increase in sucrase activity, when cultured in the presence of mesenchyme [67]. Normal development and differentiation of the small intestinal epithelium therefore depends on the interaction with mesenchyme [67] and other constituents [60].

Long-term cultures of normal primary human epithelial cells isolated from the small intestine are now well established. Human epithelial “mini-guts” can be grown from intestinal crypts and single stem cells [60, 68]. It is possible to propagate the organoid cultures in vitro that are derived from mouse [69] as well as human intestine [60]. These organoids can be grown indefinitely and display all hallmarks of the small intestinal epithelium in terms of architecture, cell types, and self-renewal characteristics [60].

Sato et al. [60] defined the conditions that promoted organoid proliferation and differentiation. Stem cells as well as organoids needed to be embedded in Matrigel which is a laminin- and collagen-rich matrix that mimics the basal lamina. Human small intestinal culture is more complex than that of mouse intestine. In addition to R-spondin, Noggin, and epidermal growth factor, the human-optimised culture conditions require more supplements (Wnt3A, gastrin, nicotinamide, inhibitor of Alk, and inhibitor of p38). Differentiation of cultured human small epithelial organoids into different cell types of the intestine requires certain supplements to be withdrawn (Wnt3, nicotinamide, and p38 inhibitor). A differentiation marker for mature enterocytes was visualised by brush border AP staining [60]. In another study, Middendorp et al. [70] also omitted certain culture supplements to achieve small intestinal differentiation of human organoids. Interestingly though, visualisation of mature enterocytes by sucrase-isomaltase staining worked well for ileal organoids but not for duodenal. Expression of lactase was induced in both ileal and duodenal organoids by so-called differentiation medium [70].

#### 3.2.3. Recent Advances in Intestinal Organoid Methods

Organoid technology might have applications in regenerative therapy for some gut diseases through ex vivo expansion of the intestinal epithelia and transplantation [71]. This methodology also enables in vitro propagation of diseased gastrointestinal tissues which might elucidate the disease pathogenesis and development of therapies [60]. Intestinal organoids have already been used to model monogenic diseases that affect the epithelium, inflammatory bowel disease (looking into cell death, mucosal integrity, and effects of inflammatory cytokines), and interactions between intestinal tissues and microbes as well as cancer ([72] and references therein). CD research lags behind these advances. It is well established that there are many cell types involved in CD pathogenesis and as such, organoids lack many of the cellular types present in an in vivo system. However, complexity can be increased through coculture with immune cells [73, 74]. Nozaki et al. [73] described the coculture of murine organoids and intraepithelial lymphocytes (IELs) in which both *αβ*T and *γδ*T cells proliferated successfully and were as motile as IELs residing in in vivo settings. Cocultures of organoids have also been described for the enteric nervous system [75] and myofibroblasts [76].

Due to advances in serological and genetic testing in diagnostic workup for celiac disease, duodenal biopsy may no longer be required for many patients. Hence, the biopsy material will become scarce. However, intestinal organoids can also be generated from embryonic stem cells or induced pluripotent stem cells ([71] and references therein) which, although they take longer to establish and are technically more demanding and costly as opposed to biopsy-derived organoids, can greatly scale up the numbers of resulting organoids and allow the clinical material instead to be used for raising the immune cells.

#### 3.2.4. Relevance of Intestinal Organoid Work and Brush Border Enzymes to the Development of State-of-the-Art In Vitro Methods for CD Research

It is well established that innate and adaptive immune responses are triggered in CD pathogenesis [77]; however, the exact sequence of these pathological events have not been elucidated to date. Organoid technology, however, allows sequential addition of the cells into the coculture, thereby enabling the study of innate immune response by reconstituting coeliac organoids and IELs separately from the effects of an adaptive immune response. In turn, gluten-specific T cells, which can also be grown in vitro, can then be included in this in vitro model of CD in order to facilitate the full toxic effects of gluten on the intestinal epithelium.

Organoid technology also enables elucidation of the role of enterocytes in the processing of gliadin peptides in CD. Enterocytes have the capacity to function as antigen-presenting cells, whereby T cell stimulation is mediated by their HLA-DR molecules [78]. Further, gliadin contains peptides that can trigger innate immune response [77, 79]. Interestingly, the gluten peptide that triggers innate immunity in coeliac disease is processed differently within the enterocyte compared to those triggering an adaptive immune response, in which the peptides reach HLA-DR positive late endosomes and are presented to lamina propria T cells [78]. Since damage to the enterocytes' brush border is one of the earliest alterations in CD, DS might be used to study the toxic effects of gluten in CD. It is important to note that brush border enzymes are an integral part of mature villous enterocytes and are quantitatively associated with their differentiation [66]. DS activity may, however, not correlate with the viability of enterocytes [80] as the brush border enzyme activities have been demonstrated in nonviable epithelial cells [81]. Hence, the immunostaining or more precisely the lack of immunostaining for brush border enzymes due to microvilli destruction rather than their activities could be used as a marker of CD pathogenesis.

Organoid technology has a substantial potential for disease modelling [82] and therefore elucidating the sequence of pathogenic events in CD in which brush border enzymes may be utilised. New in vitro models based on recent advances in stem cell research may facilitate the development of new therapeutic strategies in CD.

## 4. Conclusions

The structure of the small intestinal mucosa and the physiological processes involved are very complex. CD increases this complexity, affecting many components of the intestine including DS. Despite the fact that DS activities used to play an important role in aiding the diagnosis of CD, application of DS activities to in vitro research in CD remains to be established. The recent advances in ex vivo growth of epithelial “mini-guts” provide an exciting new platform that has become available. It enables applications to study normal or diseased epithelium with tissue engineering. Epithelial cells grow and differentiate under defined conditions which include the presence of an extracellular matrix and growth factors. A self-renewing population of epithelial cells express brush border enzymes that might be employed in the in vitro studies of CD when there is reconstitution of other immune cells into the model.

## Conflicts of Interest

The authors declare that they have no conflicts of interests.
